# Papillary thyroid carcinoma and laryngeal squamous cell carcinoma manifesting as a collision tumor of the neck: A case report

**DOI:** 10.3892/ol.2013.1628

**Published:** 2013-10-15

**Authors:** XIN WANG, XIANG-YAN CUI, NING FANG, WEI-LUN CHEN, HONG YU, WEI ZHU

**Affiliations:** Department of Otolaryngology Head-Neck Surgery, The First Hospital of Jilin University, Changchun, Jilin 130021, P.R. China

**Keywords:** laryngeal squamous cell carcinoma, papillary thyroid carcinoma, collision tumor

## Abstract

A 55-year-old male presented with a rapidly expanding mass on the right side of the neck and progressive hoarseness. An electronic laryngoscopy and a computed tomography scan were performed, and the patient was subsequently diagnosed with tumors of the larynx and the thyroid gland. An en bloc near-total thyroidectomy combined with a total laryngectomy was performed. The final pathological analysis revealed a collision tumor that was derived from a laryngeal squamous cell carcinoma and a papillary thyroid carcinoma. Collision tumors of the head and neck are rare. The therapy for a collision tumor should consist of a combination of the treatments that are normally used for each focus.

## Introduction

Although double cancers in the upper aerodigestive tract mucosa are not uncommon (1–6), collision tumors that are composed of a papillary thyroid carcinoma and a laryngeal squamous cell carcinoma are rare. The term ‘collision tumor’ refers to the coexistence of two histologically distinct malignant tumors within the same mass. The present study describes the case of a 55-year-old male who presented with a collision tumor in the neck. Written informed consent was obtained from the patient.

## Case report

**Figure 1:**
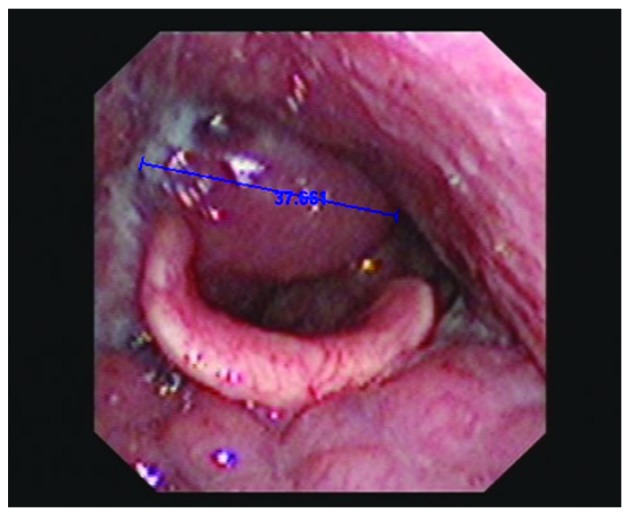
Electronic laryngoscopy image showing a large submucosal mass in the right false vocal cord and right arytenoids (diameter, 37.661 mm).

**Figure 2:**
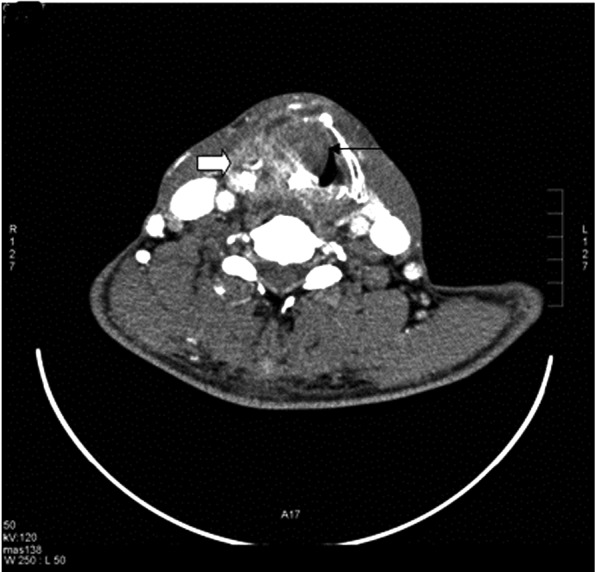
Computed tomography (CT) scan showing a thyroid carcinoma and a laryngeal carcinoma.

A 55-year-old male presented with a two-year history of a rapidly expanding, painless mass in the right side of the neck, a two-month history of progressive hoarseness and a one-month history of dyspnea. The patient had a 20-year history of smoking cigarettes and ingesting alcohol. The patient denied any loss of weight or appetite, a history of exposure to radiation or any family history of thyroid cancer. A physical examination revealed a firm, immovable 3.0×3.0×2.0-cm mass in the right side of the neck near the sternocleidomastoid muscle and a firm, fixed 1.0×1.0-cm mass in the right thyroid gland. An electronic laryngoscopy revealed a paralyzed right true vocal cord and right arytenoid, with a large submucosal mass located in the right false vocal cord and right arytenoids (Fig. 1). A computed tomography (CT) scan (Fig. 2) revealed a mass in the right parapharyngeal space, infiltrating into the right lobe of the thyroid gland, and enlarged lymph nodes in the right side of the neck. During surgery, a frozen pathological analysis demonstrated that the large submucosal mass in the right arytenoid was a laryngeal squamous cell carcinoma. The patient underwent en bloc near-total thyroidectomy combined with a total laryngectomy, as well as paratracheal lymph node and bilateral selective neck dissections (levels II–IV). The pharyngoesophageal segment was reconstructed primarily. A tracheoesophageal puncture was performed at the time of tumor resection. The patient was discharged on post-operative day 14.

**Figure 3:**
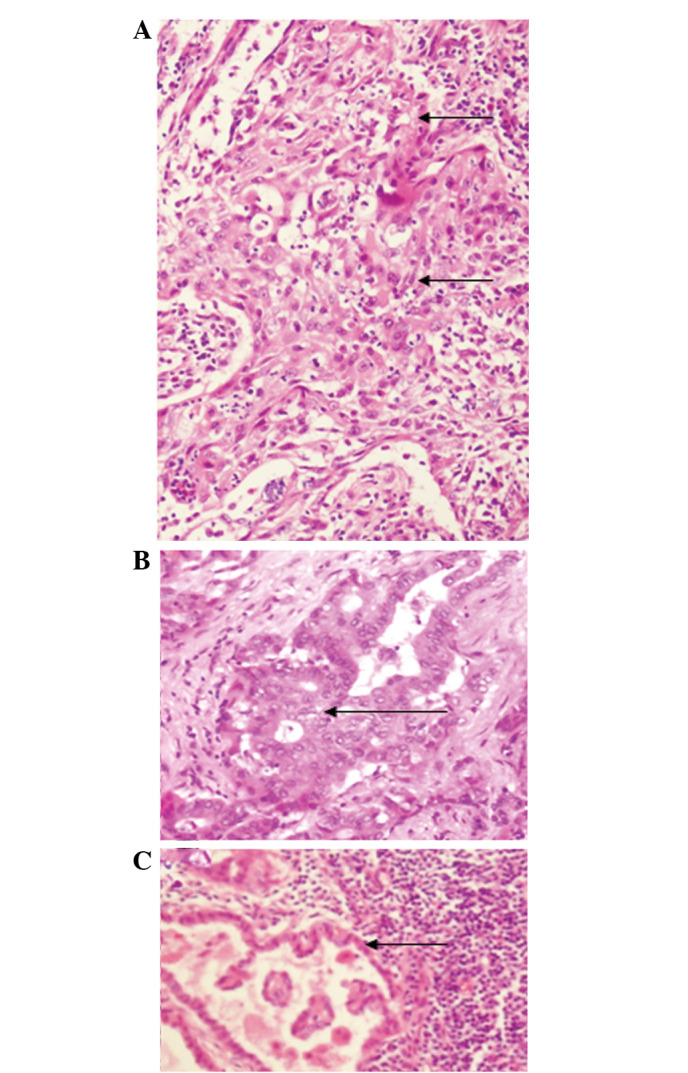
Histopathology showing (A) a laryngeal squamous cell carcinoma, (B) a papillary thyroid carcinoma and (C) lymph nodes containing metastases from the thyroid papillary carcinoma (H&E; magnification, ×200).

The resected laryngeal specimen revealed a large exophytic mass involving the entire right hemilarynx in continuity with the thyroid lesion. The final pathological analysis revealed a laryngeal squamous cell carcinoma (Fig. 3A) with infiltration of the full-thickness wall of the larynx, invasion and penetration of the thyroid cartilage and invasion of the thyroid gland. The right lobe of the thyroid gland contained a papillary thyroid carcinoma (Fig. 3B) with invasion and penetration of the thyroid cartilage, as far as the deep tissue of the larynx. Sectioning of multiple cervical lymph nodes revealed metastases from the thyroid papillary carcinoma (Fig. 3C). The final diagnosis was a collision tumor originating from a papillary thyroid carcinoma and a laryngeal squamous cell carcinoma.

**Figure 4:**
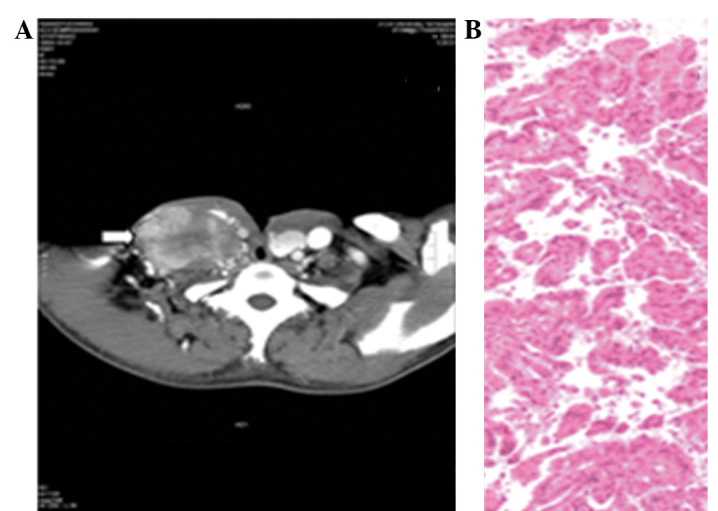
(A) Computed tomography (CT) scan showing a metastatic mass of the right neck. (B) Histopathology showing lymph nodes with metastatic papillary carcinoma (H&E; magnification, ×200).

The patient was recommended to start radiotherapy at one month post-surgery, but refused. One year after the surgery, the patient presented with a one-month history of a rapidly expanding painless mass in the right side of the neck. A physical examination revealed a firm, fixed 7.0×6.0-cm mass on the right of the tracheostomy. A CT scan (Fig. 4A) revealed a space containing a lesion on the right side of the neck that was suspected to be lymph nodes with metastatic disease. The neoplasm was removed and the pathological analysis revealed lymph nodes with metastatic papillary carcinoma (Fig. 4B). Immunohistochemistry demonstrated that the lesion was positive for cytokeratin 7, transglutaminase and thyroid transcription factor-1 and negative for galectin-3. One month after the second surgery, the patient was administered ^131^I radiation therapy for 3 days. One year after radiation therapy, the patient presented with a mass on the right side of the next and refused further therapy. Following this point the patient was lost to follow-up.

## Discussion

In multiple primary cancers, each tumor is malignant and is of an independent pathological type, with none of the lesions being metastatic (6). Multiple primary cancers may be double (i.e. two primary cancers) or triple (i.e. three primary cancers) cancers. Collision carcinomas are a special type of multiple primary carcinoma, which are difficult to diagnose prior to a surgical resection due to a lack of characteristic clinical features. In the patient of the present study, the mass presented as submucosal lesions. Since the mucosa covering the lesion remained intact, a biopsy was not performed prior to the surgery. The initial findings indicated a thyroid gland tumor that was invaded by a laryngeal tumor, or a laryngeal tumor that was invaded by a thyroid tumor. Since the frozen pathology revealed only a squamous cell carcinoma, the possibility of a collision tumor was not considered. The final pathological findings following the surgery were of a laryngeal squamous cell carcinoma and a papillary thyroid carcinoma that had invaded each other.

Collision tumors may be located anywhere in the body. A collision tumor of the breast has been described (8), as has an intracranial collision metastasis (9). Similar to the present case, a collision tumor of a papillary thyroid carcinoma and a laryngeal squamous cell carcinoma has been previously reported (7). In that patient, however, the metastatic lymph nodes were derived from a primary thyroid papillary carcinoma or a laryngeal squamous cell carcinoma, with one lymph node showing metastases from the two. In the present patient, the metastatic lymph nodes were all derived from the primary thyroid papillary carcinoma. The earlier study patient underwent a total thyroidectomy, total laryngectomy and bilateral selective neck dissections (levels II–IV), whereas the present patient underwent a subtotal thyroidectomy, total laryngectomy and bilateral selective neck dissections (levels II–IV). During the surgery, the tumor in the thyroid was considered to be derived from the larynx. Therefore, a section of the thyroid gland was preserved to ensure its continued function. The earlier study patient underwent post-operative adjuvant radiotherapy and treatment with ^131^I treatment, whereas the patient of the present study refused the two treatment modalities, perhaps for economic reasons. One year later, however, the present patient presented with cervical lymph nodes with a metastatic papillary thyroid carcinoma and underwent post-operative ^131^I treatment.

Due to the rarity of collision tumors of the head and neck, it is difficult to determine their etiology. Two hypotheses have been suggested (5). The first suggests that the two primary tumors developed in the same location by chance, perhaps due to radiation. The second hypothesis suggests that the presence of the first tumor alters the microenvironment, allowing the second, adjacent tumor to develop. The present patient and the earlier study patient were diagnosed with a collision tumor of a papillary thyroid carcinoma and a laryngeal squamous cell carcinoma (7), and the tumors were extremely large. Had these patients felt uncomfortable and gone to a hospital sooner, they may not have developed collision tumors.

The therapy for multiple primary cancers should consist of a combination of the treatments that are normally used for each focus. Since few patients with these tumors undergo a pre-operative histological diagnosis, there may be differences in the post-operative patient management. A collision carcinoma is a special type of multiple primary carcinoma. Thus, en bloc resection of the two inter-infiltrating tumors should be performed. The present patient underwent an en bloc near-total thyroidectomy combined with a total laryngectomy, along with paratracheal lymph node dissections and bilateral selective neck dissections at levels II–IV. Although post-operative adjuvant radiation therapy for the laryngeal carcinoma and ^131^I treatment for the papillary thyroid carcinoma was suggested, the patient refused further treatment. The subsequent history of the patient demonstrated that this adjuvant therapy was crucial.

Although the present patient did not exhibit any pre-operative characteristics that indicated a collision tumor rather than a laryngeal carcinoma and a thyroid carcinoma, the results indicate that patients with large tumors invading other structures should be assessed by intraoperative frozen pathology to establish a diagnosis. Although the rarity of these patients limits the therapeutic options, the results of the present study indicate that a resection should be followed by radiation therapy for laryngeal carcinoma and ^131^I treatment for the papillary thyroid carcinoma.

In conclusion, as collision tumors of the head and neck are rare, it is very difficult to obtain a pre-operative diagnosis. The therapy for a collision tumor should consist of a combination of the treatments that are normally used for each focus.
